# (*E*)-1-Methyl-4-[2-(2-naphth­yl)vin­yl]pyridinium iodide^1^

**DOI:** 10.1107/S1600536809019114

**Published:** 2009-05-29

**Authors:** Hoong-Kun Fun, Kullapa Chanawanno, Suchada Chantrapromma

**Affiliations:** a X-ray Crystallography Unit, School of Physics, Universiti Sains Malaysia, 11800 USM, Penang, Malaysia; b Crystal Materials Research Unit, Department of Chemistry, Faculty of Science, Prince of Songkla University, Hat-Yai, Songkhla 90112, Thailand

## Abstract

In the title compound, C_18_H_16_N^+^·I^−^, the cation is disordered over two orientations related by a 180° rotation about its long axis with occupancies of 0.554 (7) and 0.446 (7). Both disorder components exist in an *E* configuration. The dihedral angle between the pyridinium ring and the naphthalene ring system is 4.7 (6)° in the major disorder component and 1.6 (8)° in the minor component. In the crystal structure, centrosymmetrically related cations are stacked along the *a* axis, with significant π–π inter­actions between the pyridinium ring and the naphthalene ring system [centroid-centroid distance = 3.442 (9) Å]. The iodide ions are located between adjacent columns of cations. The cations are linked to the iodide ions by C—H⋯I inter­actions. Weak C—H⋯π inter­actions involving the methyl group are also observed.

## Related literature

For bond-length data, see: Allen *et al.* (1987 ▶). For background to non-linear optical materials research, see: Cheng *et al.* (1991*a* ▶,*b* ▶); Ogawa *et al.* (2008 ▶); Yang *et al.* (2007 ▶). For related structures, see: Chanawanno *et al.* (2008 ▶); Chantrapromma *et al.* (2006 ▶; 2007 ▶; 2008 ▶; 2009*a* ▶,*b* ▶). For the stability of the temperature controller used in the data collection, see: Cosier & Glazer (1986 ▶).
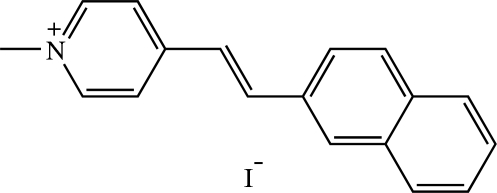


## Experimental

### 

#### Crystal data

C_18_H_16_N^+^·I^−^
*M* *_r_* = 373.29
Monoclinic, 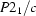

*a* = 7.2789 (1) Å
*b* = 10.9363 (2) Å
*c* = 20.0883 (4) Å
β = 101.280 (1)°
*V* = 1568.22 (5) Å^3^
*Z* = 4
Mo *K*α radiation
μ = 2.03 mm^−1^
*T* = 100 K
0.53 × 0.30 × 0.09 mm

#### Data collection

Bruker APEXII CCD area-detector diffractometer
Absorption correction: multi-scan (*SADABS*; Bruker, 2005 ▶) *T*_min_ = 0.412, *T*_max_ = 0.838
34203 measured reflections
6896 independent reflections
5373 reflections with *I* > 2σ(*I*)
*R*_int_ = 0.028

#### Refinement

*R*[*F* ^2^ > 2σ(*F* ^2^)] = 0.056
*wR*(*F* ^2^) = 0.148
*S* = 1.06
6896 reflections
338 parameters
91 restraints
H-atom parameters constrained
Δρ_max_ = 3.51 e Å^−3^
Δρ_min_ = −2.42 e Å^−3^

### 

Data collection: *APEX2* (Bruker, 2005 ▶); cell refinement: *SAINT* (Bruker, 2005 ▶); data reduction: *SAINT*; program(s) used to solve structure: *SHELXTL* (Sheldrick, 2008 ▶); program(s) used to refine structure: *SHELXTL*; molecular graphics: *SHELXTL*; software used to prepare material for publication: *SHELXTL* and *PLATON* (Spek, 2009 ▶).

## Supplementary Material

Crystal structure: contains datablocks global, I. DOI: 10.1107/S1600536809019114/ci2807sup1.cif

Structure factors: contains datablocks I. DOI: 10.1107/S1600536809019114/ci2807Isup2.hkl

Additional supplementary materials:  crystallographic information; 3D view; checkCIF report

## Figures and Tables

**Table 1:** Hydrogen-bond geometry (Å, °)

*D*—H⋯*A*	*D*—H	H⋯*A*	*D*⋯*A*	*D*—H⋯*A*
C18*A*—H18*A*⋯I1^i^	0.96	3.05	3.928 (17)	152
C18*A*—H18*B*⋯*Cg*1^i^	0.96	2.63	3.513 (18)	153
C18*A*—H18*B*⋯*Cg*2^i^	0.96	2.65	3.517 (18)	150
C18*B*—H18*E*⋯*Cg*1^i^	0.96	2.62	3.44 (2)	143
C18*B*—H18*E*⋯*Cg*2^i^	0.96	2.66	3.45 (2)	139

Symmetry code: (i) 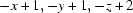. *Cg*1 and *Cg*2 are centroids of the C10*A*–C15*A* and C10*B*–C15*B* rings, respectively.

**Fig. 1.**
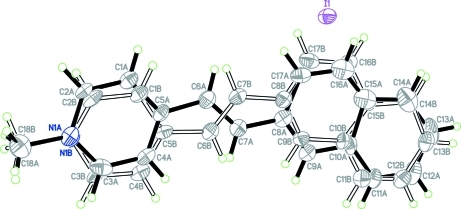
The molecular structure of the title compound, with 50% probability displacement ellipsoids and the atom-numbering scheme. Open bonds show the minor disorder component.

**Fig. 2.**
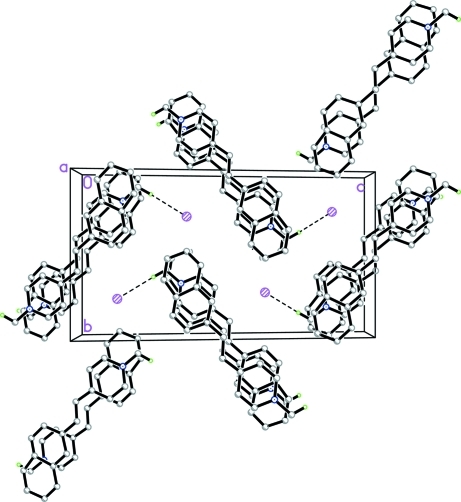
The crystal packing of the major component of the title compound viewed down the a axis. H atoms not involved in C—H···I interactions (dashed lines) have been omitted for clarity.

## supplementary crystallographic information

### Comment

In order to obtain second-order non-linear optical (NLO) single crystals, the main requirements should be the choice of molecules with large hyperpolarizability (*β*) and these molecules should align into a noncentrosymmetric space group in the crystal. Organic crystals with extensive conjugated π systems with large hyperpolarizability which exhibit NLO properties have been reported (Ogawa *et al.*, 2008; Yang *et al.*, 2007). Styryl pyridinium derivatives are considered to be good conjugated π-systems (Cheng *et al.*, 1991*a*, 1991*b*). In our on-going research in searching for NLO materials (Chanawanno *et al.*, 2008; Chantrapromma *et al.*, 2006, 2007, 2008, 2009*a*, *b*), we have previously reported the crystal structure of (*E*)-1-methyl-4-[2-(1-naphthyl)vinyl]pyridinium 4-bromobenzenesulfonate (Chantrapromma *et al.*, 2009*a*). In order to study the effect of different positions of the subsituent group and anions, the title compound was synthesized by replacing the 1-naphthyl and 4-bromobenzenesulfonate in the (*E*)-1-methyl-4-[2-(1-naphthyl)vinyl]pyridinium 4-bromobenzenesulfonate with 2-naphthyl and iodide in the title compound. However it crystallized in the monoclinic centrosymmetric space group *P*2_1_/*c* and would not exhibit second-order nonlinear optical properties.

Fig. 1 shows the asymmetric unit of the title compound which consists of a C_18_H_16_N^+^ cation and a I^-^ anion. The whole cation is disordered over two sites; the major component *A* and the minor component *B* (Fig. 1), with the refined site-occupancy ratio of 0.554 (7)/0.446 (7). The cation exists in the *E* configuration with respect to the C6═C7 double bond. The napthalenyl moiety is essentially planar in both disorder components as indicated by the interplanar angle between the two aromatic C8-C10/C15-C17 and C10–C15 rings [1.5 (8)° for the major component *A* and 3.2 (9)° for the minor component *B*]. The major component *A* of cation is slightly twisted with the dihedral angle between the pyridinium and the mean plane through the napthalenyl moiety (C8–C17) being 4.7 (6)° whereas the minor component *B* is almost planar [dihedral angle 1.6 (8)°]. The C4–C5–C6–C7 and C6–C7–C8–C17 torsion angles [0.4 (10)° and 2.1 (10)° in the major component and -179.4 (8)° and 179.9 (8)° in the minor component] in both disorder components indicate that the orientations of the ethynyl moiety in these components are related by 180° rotation about the long axis of the molecule. The bond lengths are in normal ranges (Allen *et al.*, 1987) and are comparable to those observed in related structures (Chanawanno *et al.*, 2008; Chantrapromma *et al.*, 2006, 2007, 2008, 2009*a*,*b*).

In the crystal packing (Fig. 2), centrosymmetrically related cations are stacked along the *a* axis, with significant π-π interactions between pyridinium ring and naphthalene ring system [centroid-centroid distance is 3.442 (9) Å]. The iodide ions are located between adjacent coloumns of cations. The cations are linked to the iodide ions by C—H···I weak interactions (Table 1). The crystal structure is further stabilized by C—H···π interactions involving the methyl group (Table 1; Cg1 and Cg2 are centroids of the C10A-C15A and C10B-C15B rings, respectively).

### Experimental

The title compound was prepared by mixing 1:1:1 molar ratio solutions of 1,4-dimethylpyridinium iodide (2 g, 8.5 mmol), 2-naphthaldehyde (1.16 ml, 8.5 mmol) and piperidine (0.84 ml, 8.5 mmol) in methanol (40 ml). The resulting solution was refluxed for 3 h under a nitrogen atmosphere. The solid compound formed was filtered and washed with diethylether. Yellow plate-shaped single crystals of the title compound suitable for *X*-ray structure determination were recrystallized from methanol by slow evaporation at room temperature over a few weeks (m.p. 557-558 K).

### Refinement

The cation is disordered over two orientations with occupancies of 0.554 (7) and 0.446 (7). The same *U*_ij_ parameters were used for atom pairs N1A/N1B and C15A/C16A and all disordered atoms were subjected to a rigid bond restraint. All H atoms were positioned geometrically and allowed to ride on their parent atoms, with C-H = 0.93 Å (aromatic and CH) and 0.96 Å (CH_3_). The *U*_iso_ values were constrained to be 1.5*U*_eq_ of the carrier atom for methyl H atoms and 1.2*U*_eq_ for the remaining H atoms. A rotating group model was used for the methyl groups. The highest residual electron density peak is located at 0.69 Å from I1 and the deepest hole is located at 0.56 Å from I1.

### Crystal data

**Table:**

C_18_H_16_N^+^·I^−^	*F*(000) = 736
*M**_r_* = 373.29	*D*_x_ = 1.581 Mg m^−^^3^
Monoclinic, *P*2_1_/*c*	Melting point = 557–558 K
Hall symbol: -P 2ybc	Mo *K*α radiation, λ = 0.71073 Å
*a* = 7.2789 (1) Å	Cell parameters from 6896 reflections
*b* = 10.9363 (2) Å	θ = 2.1–35.0°
*c* = 20.0883 (4) Å	µ = 2.03 mm^−^^1^
β = 101.280 (1)°	*T* = 100 K
*V* = 1568.22 (5) Å^3^	Plate, yellow
*Z* = 4	0.53 × 0.30 × 0.09 mm

### Data collection

**Table:**

Bruker APEXII CCD area-detector diffractometer	6896 independent reflections
Radiation source: sealed tube	5373 reflections with *I* > 2σ(*I*)
graphite	*R*_int_ = 0.028
φ and ω scans	θ_max_ = 35.0°, θ_min_ = 2.1°
Absorption correction: multi-scan (*SADABS*; Bruker, 2005)	*h* = −11→11
*T*_min_ = 0.412, *T*_max_ = 0.838	*k* = −17→16
34203 measured reflections	*l* = −32→27

### Refinement

**Table:**

Refinement on *F*^2^	Primary atom site location: structure-invariant direct methods
Least-squares matrix: full	Secondary atom site location: difference Fourier map
*R*[*F*^2^ > 2σ(*F*^2^)] = 0.056	Hydrogen site location: inferred from neighbouring sites
*wR*(*F*^2^) = 0.148	H-atom parameters constrained
*S* = 1.06	*w* = 1/[σ^2^(*F*_o_^2^) + (0.0586*P*)^2^ + 4.6136*P*] where *P* = (*F*_o_^2^ + 2*F*_c_^2^)/3
6896 reflections	(Δ/σ)_max_ = 0.001
338 parameters	Δρ_max_ = 3.51 e Å^−^^3^
91 restraints	Δρ_min_ = −2.42 e Å^−^^3^

### Special details

**Table:**

Experimental. The crystal was placed in the cold stream of an Oxford Cryosystems Cobra open-flow nitrogen cryostat (Cosier & Glazer, 1986) operating at 100.0 (1) K.
Geometry. All esds (except the esd in the dihedral angle between two l.s. planes) are estimated using the full covariance matrix. The cell esds are taken into account individually in the estimation of esds in distances, angles and torsion angles; correlations between esds in cell parameters are only used when they are defined by crystal symmetry. An approximate (isotropic) treatment of cell esds is used for estimating esds involving l.s. planes.
Refinement. Refinement of F^2^ against ALL reflections. The weighted R-factor wR and goodness of fit S are based on F^2^, conventional R-factors R are based on F, with F set to zero for negative F^2^. The threshold expression of F^2^ > 2sigma(F^2^) is used only for calculating R-factors(gt) etc. and is not relevant to the choice of reflections for refinement. R-factors based on F^2^ are statistically about twice as large as those based on F, and R- factors based on ALL data will be even larger.

### Fractional atomic coordinates and isotropic or equivalent isotropic displacement parameters (Å^2^)

**Table:**

	*x*	*y*	*z*	*U*_iso_*/*U*_eq_	Occ. (<1)
I1	0.48129 (4)	0.73286 (2)	0.633723 (12)	0.03874 (9)	
N1A	0.520 (4)	0.174 (3)	1.153 (2)	0.022 (2)	0.555 (7)
C1A	0.3651 (10)	0.1993 (7)	1.0385 (4)	0.0268 (12)	0.555 (7)
H1AA	0.3113	0.1634	0.9973	0.032*	0.555 (7)
C2A	0.452 (2)	0.1269 (8)	1.0898 (7)	0.0230 (16)	0.555 (7)
H2AA	0.4665	0.0440	1.0820	0.028*	0.555 (7)
C3A	0.516 (2)	0.2966 (15)	1.1600 (7)	0.033 (2)	0.555 (7)
H3AA	0.5689	0.3307	1.2018	0.039*	0.555 (7)
C4A	0.4359 (12)	0.3744 (8)	1.1076 (5)	0.0290 (15)	0.555 (7)
H4AA	0.4382	0.4586	1.1145	0.035*	0.555 (7)
C5A	0.3533 (8)	0.3270 (6)	1.0454 (4)	0.0238 (11)	0.555 (7)
C6A	0.2561 (8)	0.4011 (5)	0.9884 (3)	0.0283 (12)	0.555 (7)
H6AA	0.2049	0.3616	0.9480	0.034*	0.555 (7)
C7A	0.2374 (8)	0.5225 (5)	0.9915 (3)	0.0282 (12)	0.555 (7)
H7AA	0.2908	0.5591	1.0325	0.034*	0.555 (7)
C8A	0.1433 (9)	0.6045 (7)	0.9383 (4)	0.0268 (11)	0.555 (7)
C9A	0.1464 (11)	0.7284 (8)	0.9532 (4)	0.0263 (12)	0.555 (7)
H9AA	0.2117	0.7573	0.9947	0.032*	0.555 (7)
C10A	0.047 (3)	0.8108 (13)	0.9036 (9)	0.028 (2)	0.555 (7)
C11A	0.0570 (16)	0.9380 (9)	0.9171 (5)	0.0303 (17)	0.555 (7)
H11A	0.1258	0.9661	0.9582	0.036*	0.555 (7)
C12A	−0.0330 (19)	1.0196 (11)	0.8709 (6)	0.041 (2)	0.555 (7)
H12A	−0.0320	1.1026	0.8811	0.049*	0.555 (7)
C13A	−0.128 (4)	0.976 (2)	0.8070 (8)	0.047 (4)	0.555 (7)
H13A	−0.1882	1.0300	0.7738	0.056*	0.555 (7)
C14A	−0.129 (4)	0.852 (2)	0.7955 (13)	0.047 (3)	0.555 (7)
H14A	−0.1968	0.8284	0.7534	0.057*	0.555 (7)
C15A	−0.0433 (17)	0.7529 (12)	0.8368 (7)	0.0400 (17)	0.555 (7)
C16A	−0.040 (2)	0.6423 (19)	0.8260 (7)	0.0400 (17)	0.555 (7)
H16A	−0.1012	0.6131	0.7840	0.048*	0.555 (7)
C17A	0.0489 (12)	0.5599 (8)	0.8729 (4)	0.0312 (14)	0.555 (7)
H17A	0.0484	0.4769	0.8628	0.037*	0.555 (7)
C18A	0.618 (2)	0.0940 (16)	1.2057 (9)	0.040 (4)	0.555 (7)
H18A	0.6436	0.1367	1.2483	0.061*	0.555 (7)
H18B	0.7343	0.0685	1.1941	0.061*	0.555 (7)
H18C	0.5423	0.0236	1.2095	0.061*	0.555 (7)
N1B	0.544 (5)	0.186 (3)	1.151 (3)	0.022 (2)	0.45
C1B	0.3643 (13)	0.2454 (8)	1.0446 (5)	0.0262 (16)	0.445 (7)
H1BA	0.3072	0.2232	1.0007	0.031*	0.445 (7)
C2B	0.443 (3)	0.1573 (12)	1.0917 (10)	0.029 (2)	0.445 (7)
H2BA	0.4241	0.0752	1.0805	0.034*	0.445 (7)
C3B	0.546 (3)	0.3028 (12)	1.1733 (8)	0.021 (2)	0.445 (7)
H3BA	0.6026	0.3218	1.2177	0.025*	0.445 (7)
C4B	0.4663 (15)	0.3915 (9)	1.1306 (5)	0.0257 (15)	0.445 (7)
H4BA	0.4740	0.4721	1.1457	0.031*	0.445 (7)
C5B	0.3727 (11)	0.3669 (8)	1.0646 (4)	0.0246 (14)	0.445 (7)
C6B	0.2907 (10)	0.4672 (7)	1.0211 (4)	0.0276 (14)	0.445 (7)
H6BA	0.3067	0.5450	1.0401	0.033*	0.445 (7)
C7B	0.1948 (10)	0.4609 (7)	0.9569 (4)	0.0277 (14)	0.445 (7)
H7BA	0.1786	0.3843	0.9366	0.033*	0.445 (7)
C8B	0.1140 (11)	0.5666 (8)	0.9168 (5)	0.0258 (14)	0.445 (7)
C9B	0.1359 (14)	0.6857 (9)	0.9432 (5)	0.0248 (16)	0.445 (7)
H9BA	0.1989	0.6971	0.9877	0.030*	0.445 (7)
C10B	0.066 (3)	0.7876 (14)	0.9045 (12)	0.024 (2)	0.445 (7)
C11B	0.073 (2)	0.9053 (11)	0.9303 (6)	0.031 (2)	0.445 (7)
H11B	0.1359	0.9193	0.9747	0.037*	0.445 (7)
C12B	−0.009 (2)	1.0020 (13)	0.8918 (6)	0.035 (2)	0.445 (7)
H12B	0.0004	1.0804	0.9101	0.042*	0.445 (7)
C13B	−0.110 (4)	0.983 (2)	0.8243 (10)	0.033 (3)	0.445 (7)
H13B	−0.1654	1.0503	0.8000	0.040*	0.445 (7)
C14B	−0.128 (5)	0.8686 (15)	0.7928 (12)	0.024 (3)	0.445 (7)
H14B	−0.1891	0.8547	0.7483	0.029*	0.445 (7)
C15B	−0.0405 (15)	0.7753 (7)	0.8384 (5)	0.0125 (13)*	0.445 (7)
C16B	−0.056 (2)	0.6447 (17)	0.8116 (6)	0.031 (2)	0.445 (7)
H16B	−0.1177	0.6311	0.7671	0.037*	0.445 (7)
C17B	0.0140 (14)	0.5504 (11)	0.8485 (5)	0.0323 (19)	0.445 (7)
H17B	−0.0016	0.4722	0.8301	0.039*	0.445 (7)
C18B	0.624 (3)	0.0915 (12)	1.2029 (8)	0.026 (3)	0.445 (7)
H18G	0.5790	0.0120	1.1874	0.039*	0.445 (7)
H18D	0.5873	0.1095	1.2451	0.039*	0.445 (7)
H18E	0.7586	0.0928	1.2093	0.039*	0.445 (7)

### Atomic displacement parameters (Å^2^)

**Table:**

	*U*^11^	*U*^22^	*U*^33^	*U*^12^	*U*^13^	*U*^23^
I1	0.05884 (17)	0.02652 (11)	0.03108 (12)	−0.00509 (9)	0.00937 (10)	0.00240 (8)
N1A	0.014 (7)	0.019 (5)	0.037 (3)	−0.005 (4)	0.014 (4)	0.003 (3)
C1A	0.029 (3)	0.021 (3)	0.033 (3)	−0.003 (3)	0.013 (2)	0.000 (3)
C2A	0.024 (3)	0.015 (4)	0.032 (3)	0.000 (3)	0.011 (2)	−0.005 (3)
C3A	0.020 (5)	0.044 (4)	0.034 (5)	−0.010 (3)	0.004 (3)	−0.011 (3)
C4A	0.030 (4)	0.022 (3)	0.037 (4)	0.002 (2)	0.012 (3)	−0.005 (3)
C5A	0.027 (3)	0.017 (3)	0.030 (3)	0.000 (2)	0.012 (2)	0.002 (2)
C6A	0.029 (3)	0.025 (2)	0.033 (3)	0.0001 (19)	0.009 (2)	0.001 (2)
C7A	0.026 (2)	0.026 (2)	0.033 (3)	−0.0007 (19)	0.007 (2)	0.000 (2)
C8A	0.028 (3)	0.025 (3)	0.030 (3)	−0.003 (2)	0.012 (2)	0.000 (2)
C9A	0.026 (3)	0.025 (3)	0.030 (3)	−0.001 (3)	0.011 (2)	0.000 (3)
C10A	0.020 (5)	0.039 (5)	0.026 (3)	0.013 (4)	0.010 (3)	0.008 (4)
C11A	0.029 (3)	0.027 (4)	0.037 (4)	0.002 (3)	0.011 (3)	0.008 (3)
C12A	0.032 (5)	0.035 (4)	0.058 (7)	0.005 (3)	0.017 (5)	0.018 (4)
C13A	0.027 (5)	0.066 (7)	0.049 (8)	0.011 (6)	0.012 (6)	0.025 (6)
C14A	0.027 (7)	0.073 (8)	0.045 (7)	−0.001 (8)	0.013 (5)	0.008 (6)
C15A	0.027 (3)	0.055 (4)	0.042 (3)	−0.009 (3)	0.015 (2)	−0.007 (3)
C16A	0.027 (3)	0.055 (4)	0.042 (3)	−0.009 (3)	0.015 (2)	−0.007 (3)
C17A	0.032 (4)	0.030 (3)	0.033 (4)	−0.008 (2)	0.011 (3)	−0.008 (3)
C18A	0.019 (5)	0.049 (7)	0.050 (7)	0.000 (4)	−0.001 (5)	−0.001 (5)
N1B	0.014 (7)	0.019 (5)	0.037 (3)	−0.005 (4)	0.014 (4)	0.003 (3)
C1B	0.030 (3)	0.017 (4)	0.033 (4)	−0.003 (3)	0.011 (3)	−0.001 (3)
C2B	0.030 (4)	0.018 (5)	0.042 (4)	0.003 (5)	0.019 (3)	−0.007 (4)
C3B	0.016 (5)	0.012 (3)	0.035 (6)	−0.005 (3)	0.004 (4)	−0.007 (3)
C4B	0.031 (4)	0.019 (3)	0.031 (4)	−0.006 (3)	0.017 (3)	−0.003 (3)
C5B	0.030 (3)	0.021 (3)	0.026 (3)	−0.003 (3)	0.012 (3)	−0.007 (2)
C6B	0.029 (3)	0.022 (3)	0.032 (3)	0.001 (2)	0.009 (2)	0.001 (2)
C7B	0.028 (3)	0.025 (3)	0.032 (3)	0.001 (2)	0.009 (2)	−0.004 (2)
C8B	0.024 (3)	0.022 (3)	0.031 (4)	0.000 (3)	0.007 (3)	0.000 (3)
C9B	0.029 (3)	0.024 (4)	0.022 (3)	0.000 (3)	0.008 (2)	−0.003 (3)
C10B	0.009 (4)	0.031 (5)	0.032 (4)	0.007 (4)	0.007 (3)	−0.004 (4)
C11B	0.031 (4)	0.032 (5)	0.030 (5)	0.000 (4)	0.008 (4)	0.002 (4)
C12B	0.036 (5)	0.032 (5)	0.039 (6)	0.003 (4)	0.014 (5)	0.000 (4)
C13B	0.026 (7)	0.037 (5)	0.041 (7)	0.006 (4)	0.015 (6)	0.005 (5)
C14B	0.018 (6)	0.028 (4)	0.026 (4)	0.007 (3)	0.003 (4)	0.015 (3)
C16B	0.030 (5)	0.029 (4)	0.035 (5)	0.002 (3)	0.009 (4)	−0.003 (4)
C17B	0.026 (4)	0.045 (5)	0.026 (4)	−0.003 (3)	0.006 (3)	−0.011 (4)
C18B	0.039 (8)	0.014 (4)	0.032 (5)	0.012 (4)	0.022 (5)	0.012 (4)

### Geometric parameters (Å, °)

**Table:**

N1A—C3A	1.34 (3)	N1B—C2B	1.31 (6)
N1A—C2A	1.38 (4)	N1B—C3B	1.36 (4)
N1A—C18A	1.45 (4)	N1B—C18B	1.50 (5)
C1A—C2A	1.354 (13)	C1B—C5B	1.385 (12)
C1A—C5A	1.408 (10)	C1B—C2B	1.393 (18)
C1A—H1AA	0.93	C1B—H1BA	0.93
C2A—H2AA	0.93	C2B—H2BA	0.93
C3A—C4A	1.390 (17)	C3B—C4B	1.350 (17)
C3A—H3AA	0.93	C3B—H3BA	0.93
C4A—C5A	1.378 (10)	C4B—C5B	1.393 (12)
C4A—H4AA	0.93	C4B—H4BA	0.93
C5A—C6A	1.467 (8)	C5B—C6B	1.456 (11)
C6A—C7A	1.337 (8)	C6B—C7B	1.342 (10)
C6A—H6AA	0.93	C6B—H6BA	0.93
C7A—C8A	1.460 (9)	C7B—C8B	1.465 (11)
C7A—H7AA	0.93	C7B—H7BA	0.93
C8A—C9A	1.386 (10)	C8B—C9B	1.404 (12)
C8A—C17A	1.442 (10)	C8B—C17B	1.432 (12)
C9A—C10A	1.429 (15)	C9B—C10B	1.397 (19)
C9A—H9AA	0.93	C9B—H9BA	0.93
C10A—C11A	1.417 (16)	C10B—C11B	1.385 (19)
C10A—C15A	1.51 (2)	C10B—C15B	1.41 (2)
C11A—C12A	1.360 (11)	C11B—C12B	1.378 (15)
C11A—H11A	0.93	C11B—H11B	0.93
C12A—C13A	1.416 (17)	C12B—C13B	1.424 (17)
C12A—H12A	0.93	C12B—H12B	0.93
C13A—C14A	1.38 (3)	C13B—C14B	1.40 (2)
C13A—H13A	0.93	C13B—H13B	0.93
C14A—C15A	1.43 (2)	C14B—C15B	1.435 (15)
C14A—H14A	0.93	C14B—H14B	0.93
C15A—C16A	1.23 (2)	C15B—C16B	1.52 (2)
C16A—C17A	1.372 (18)	C16B—C17B	1.313 (19)
C16A—H16A	0.93	C16B—H16B	0.93
C17A—H17A	0.93	C17B—H17B	0.93
C18A—H18A	0.96	C18B—H18G	0.96
C18A—H18B	0.96	C18B—H18D	0.96
C18A—H18C	0.96	C18B—H18E	0.96
			
C3A—N1A—C2A	117 (3)	C5B—C1B—C2B	118.5 (10)
C3A—N1A—C18A	123 (3)	C5B—C1B—H1BA	120.7
C2A—N1A—C18A	119 (2)	C2B—C1B—H1BA	120.7
C2A—C1A—C5A	122.2 (7)	N1B—C2B—C1B	123 (2)
C2A—C1A—H1AA	118.9	N1B—C2B—H2BA	118.7
C5A—C1A—H1AA	118.9	C1B—C2B—H2BA	118.7
C1A—C2A—N1A	120.9 (15)	C4B—C3B—N1B	120 (2)
C1A—C2A—H2AA	119.5	C4B—C3B—H3BA	120.2
N1A—C2A—H2AA	119.5	N1B—C3B—H3BA	120.2
N1A—C3A—C4A	123 (2)	C3B—C4B—C5B	122.4 (9)
N1A—C3A—H3AA	118.4	C3B—C4B—H4BA	118.8
C4A—C3A—H3AA	118.4	C5B—C4B—H4BA	118.8
C5A—C4A—C3A	120.0 (9)	C1B—C5B—C4B	116.6 (8)
C5A—C4A—H4AA	120.0	C1B—C5B—C6B	124.0 (8)
C3A—C4A—H4AA	120.0	C4B—C5B—C6B	119.5 (8)
C4A—C5A—C1A	116.0 (6)	C7B—C6B—C5B	127.8 (7)
C4A—C5A—C6A	123.9 (7)	C7B—C6B—H6BA	116.1
C1A—C5A—C6A	120.0 (7)	C5B—C6B—H6BA	116.1
C7A—C6A—C5A	123.4 (6)	C6B—C7B—C8B	124.5 (7)
C7A—C6A—H6AA	118.3	C6B—C7B—H7BA	117.8
C5A—C6A—H6AA	118.3	C8B—C7B—H7BA	117.8
C6A—C7A—C8A	127.9 (6)	C9B—C8B—C17B	118.4 (8)
C6A—C7A—H7AA	116.1	C9B—C8B—C7B	121.3 (8)
C8A—C7A—H7AA	116.1	C17B—C8B—C7B	120.3 (9)
C9A—C8A—C17A	120.8 (6)	C10B—C9B—C8B	121.8 (11)
C9A—C8A—C7A	117.2 (7)	C10B—C9B—H9BA	119.1
C17A—C8A—C7A	122.0 (7)	C8B—C9B—H9BA	119.1
C8A—C9A—C10A	118.7 (10)	C11B—C10B—C9B	123.3 (17)
C8A—C9A—H9AA	120.6	C11B—C10B—C15B	114.6 (13)
C10A—C9A—H9AA	120.6	C9B—C10B—C15B	121.6 (13)
C11A—C10A—C9A	119.1 (14)	C12B—C11B—C10B	121.5 (13)
C11A—C10A—C15A	125.2 (12)	C12B—C11B—H11B	119.3
C9A—C10A—C15A	115.3 (11)	C10B—C11B—H11B	119.3
C12A—C11A—C10A	121.0 (12)	C11B—C12B—C13B	120.7 (13)
C12A—C11A—H11A	119.5	C11B—C12B—H12B	119.7
C10A—C11A—H11A	119.5	C13B—C12B—H12B	119.7
C11A—C12A—C13A	119.0 (13)	C14B—C13B—C12B	123.1 (17)
C11A—C12A—H12A	120.5	C14B—C13B—H13B	118.4
C13A—C12A—H12A	120.5	C12B—C13B—H13B	118.4
C14A—C13A—C12A	117.7 (18)	C13B—C14B—C15B	110.9 (17)
C14A—C13A—H13A	121.1	C13B—C14B—H14B	124.6
C12A—C13A—H13A	121.1	C15B—C14B—H14B	124.6
C13A—C14A—C15A	132 (2)	C10B—C15B—C14B	129.0 (13)
C13A—C14A—H14A	114.2	C10B—C15B—C16B	114.3 (9)
C15A—C14A—H14A	114.2	C14B—C15B—C16B	116.6 (12)
C16A—C15A—C14A	131.7 (17)	C17B—C16B—C15B	122.9 (12)
C16A—C15A—C10A	123.1 (14)	C17B—C16B—H16B	118.5
C14A—C15A—C10A	105.1 (14)	C15B—C16B—H16B	118.5
C15A—C16A—C17A	123.3 (14)	C16B—C17B—C8B	120.8 (11)
C15A—C16A—H16A	118.4	C16B—C17B—H17B	119.6
C17A—C16A—H16A	118.4	C8B—C17B—H17B	119.6
C16A—C17A—C8A	118.6 (10)	N1B—C18B—H18G	109.5
C16A—C17A—H17A	120.7	N1B—C18B—H18D	109.5
C8A—C17A—H17A	120.7	H18G—C18B—H18D	109.5
C2B—N1B—C3B	120 (3)	N1B—C18B—H18E	109.5
C2B—N1B—C18B	123 (3)	H18G—C18B—H18E	109.5
C3B—N1B—C18B	116 (4)	H18D—C18B—H18E	109.5
			
C5A—C1A—C2A—N1A	6(2)	C3B—N1B—C2B—C1B	12 (4)
C3A—N1A—C2A—C1A	−8(3)	C18B—N1B—C2B—C1B	178 (2)
C18A—N1A—C2A—C1A	−177.7 (16)	C5B—C1B—C2B—N1B	−8(3)
C2A—N1A—C3A—C4A	4(3)	C2B—N1B—C3B—C4B	−9(4)
C18A—N1A—C3A—C4A	174.0 (18)	C18B—N1B—C3B—C4B	−176.2 (19)
N1A—C3A—C4A—C5A	1(3)	N1B—C3B—C4B—C5B	3(3)
C3A—C4A—C5A—C1A	−3.3 (14)	C2B—C1B—C5B—C4B	2.0 (16)
C3A—C4A—C5A—C6A	176.4 (11)	C2B—C1B—C5B—C6B	−178.1 (12)
C2A—C1A—C5A—C4A	0.1 (13)	C3B—C4B—C5B—C1B	0.3 (17)
C2A—C1A—C5A—C6A	−179.6 (10)	C3B—C4B—C5B—C6B	−179.6 (13)
C4A—C5A—C6A—C7A	−0.4 (10)	C1B—C5B—C6B—C7B	0.7 (13)
C1A—C5A—C6A—C7A	179.2 (6)	C4B—C5B—C6B—C7B	−179.4 (8)
C5A—C6A—C7A—C8A	−179.7 (6)	C5B—C6B—C7B—C8B	179.0 (7)
C6A—C7A—C8A—C9A	−178.5 (6)	C6B—C7B—C8B—C9B	1.5 (12)
C6A—C7A—C8A—C17A	2.1 (10)	C6B—C7B—C8B—C17B	179.9 (8)
C17A—C8A—C9A—C10A	2.7 (14)	C17B—C8B—C9B—C10B	−1.0 (18)
C7A—C8A—C9A—C10A	−176.7 (12)	C7B—C8B—C9B—C10B	177.5 (14)
C8A—C9A—C10A—C11A	−177.1 (13)	C8B—C9B—C10B—C11B	175.4 (16)
C8A—C9A—C10A—C15A	−4(2)	C8B—C9B—C10B—C15B	4(3)
C9A—C10A—C11A—C12A	179.0 (13)	C9B—C10B—C11B—C12B	−175.2 (17)
C15A—C10A—C11A—C12A	6(3)	C15B—C10B—C11B—C12B	−3(3)
C10A—C11A—C12A—C13A	−4(2)	C10B—C11B—C12B—C13B	1(3)
C11A—C12A—C13A—C14A	2(4)	C11B—C12B—C13B—C14B	−1(4)
C12A—C13A—C14A—C15A	−2(5)	C12B—C13B—C14B—C15B	2(4)
C13A—C14A—C15A—C16A	−178 (3)	C11B—C10B—C15B—C14B	6(3)
C13A—C14A—C15A—C10A	4(4)	C9B—C10B—C15B—C14B	178 (2)
C11A—C10A—C15A—C16A	176.1 (17)	C11B—C10B—C15B—C16B	−177.2 (17)
C9A—C10A—C15A—C16A	3(2)	C9B—C10B—C15B—C16B	−5(3)
C11A—C10A—C15A—C14A	−6(3)	C13B—C14B—C15B—C10B	−5(4)
C9A—C10A—C15A—C14A	−178.7 (19)	C13B—C14B—C15B—C16B	178 (2)
C14A—C15A—C16A—C17A	−179 (2)	C10B—C15B—C16B—C17B	4(2)
C10A—C15A—C16A—C17A	−1(2)	C14B—C15B—C16B—C17B	−178 (2)
C15A—C16A—C17A—C8A	0(2)	C15B—C16B—C17B—C8B	−1(2)
C9A—C8A—C17A—C16A	−0.9 (13)	C9B—C8B—C17B—C16B	−0.2 (16)
C7A—C8A—C17A—C16A	178.5 (10)	C7B—C8B—C17B—C16B	−178.7 (12)

### Hydrogen-bond geometry (Å, °)

**Table:**

*D*—H···*A*	*D*—H	H···*A*	*D*···*A*	*D*—H···*A*
C18A—H18A···I1^i^	0.96	3.05	3.928 (17)	152
C18A—H18B···Cg1^i^	0.96	2.63	3.513 (18)	153
C18A—H18B···Cg2^i^	0.96	2.65	3.517 (18)	150
C18B—H18E···Cg1^i^	0.96	2.62	3.44 (2)	143
C18B—H18E···Cg2^i^	0.96	2.66	3.45 (2)	139

Symmetry codes: (i) −*x*+1, −*y*+1, −*z*+2.
